# Survey of *Candidatus* Liberibacter Solanacearum and Its Associated Vectors in Potato Crop in Spain

**DOI:** 10.3390/insects13100964

**Published:** 2022-10-21

**Authors:** M. Carmen Asensio-S.-Manzanera, Yolanda Santiago-Calvo, José Luis Palomo-Gómez, Raquel Marquínez-Ramírez, Saskia Bastin, Eva María García-Méndez, Estrella Hernández-Suárez, Felipe Siverio-de-la-Rosa

**Affiliations:** 1 Instituto Tecnológico Agrario de Castilla y León (ITACyL), Junta de Castilla y León, 47071 Valladolid, Spain; 2 Centro Regional de Diagnóstico, Junta Castilla y León, 37340 Aldearrubia, Spain; 3 Laboratorio de Analíticas Vegetales NEIKER_BRTA, 01192 Arkaute, Spain; 4 Instituto Canario de Investigaciones Agrarias (ICIA), 38270 San Cristóbal de La Laguna, Spain; 5 Centro de Investigación y Formación Agrarias (CIFA), 39600 Muriedas, Spain

**Keywords:** *Bactericera nigricornis*, zebra chip, psyllid, transmission, distribution

## Abstract

**Simple Summary:**

Zebra chip disease (ZC) in potato is an economically important disorder, documented in North and Central America and New Zealand. It is associated with the bacterium ‘*Candidatus* Liberibacter solanacearum’ (CaLsol), which is transmitted by the psyllid *Bactericera cockerelli*. The characteristic symptom of ZC is a dark and light striped pattern in chips, making them commercially unacceptable. Naturally CaLsol-infected potato tubers were reported in Europe, where the threat of ZC for European potato crops is a major concern. This work studied the presence and abundance of ZC symptoms and CaLsol in potato plants, as well as those psyllid species that eventually could transmit the bacterium, in the main producing areas in Spain. Very few symptomatic and CaLsol-positive plants were detected in Mainland Spain, and any positive plant was detected in the Canary Islands. Most of the adult psyllids captured were identified as *Bactericera nigricornis*, and some of them as *Bactericera trigonica*, but no *B. cockerelli* was detected. *B. nigricornis* was found widely distributed in the northern half of the Iberian Peninsula, and it was the only one able to feed and reproduce on potato plants. However, this psyllid does not seem sufficient to pose a threat to potato production, due to the scarce number of specimens and because the frequency of *B. nigricornis* specimens that were CaLsol+ was very low.

**Abstract:**

‘*Candidatus* Liberibacter solanacearum’ (CaLsol), the etiological agent of potato zebra chip (ZC), is transmitted to potato plants by the psyllid *Bactericera cockerelli* (Šulc, 1909) in North and Central America and New Zealand. The risk of the dispersion of ZC in Spain depends on the presence of an efficient vector. This work studies the presence and abundance of ZC symptoms and CaLsol in potato plants, as well as the presence and abundance of psyllid species associated with potato crops in the main producing areas in Spain. Eighty-eight plots were surveyed punctually to detect ZC symptoms and psyllid species in the main potato-producing areas. Furthermore, fourteen potato plots were surveyed by different sampling methods during the cropping season to detect psyllid species from 2016 to 2018. Very few symptomatic and CaLsol-positive plants were detected in Mainland Spain, and any positive plant was detected in the Canary Islands. Most of the adult psyllids captured were identified as *Bactericera nigricornis* (Foerster, 1848), and some of them as *Bactericera trigonica*, but no *B. cockerelli* was detected. *B. nigricornis* was found widely distributed in the northern half of the Iberian Peninsula; however, this psyllid does not seem sufficient to pose a threat to potato production, due to the scarce number of specimens and because the frequency of *B. nigricornis* specimens that were CaLsol+ was very low.

## 1. Introduction

*Candidatus* Liberibacter solanacearum (CaLsol) is a Gram-negative bacterium living as a phloem-limited obligate parasite in plants and is associated with zebra chip disease (ZC) in potato. This is an economically important disorder, documented in commercial fields in North and Central America and New Zealand [1]. In the USA, potato yield losses were estimated to reach up to 100% for farmers [2], and chemical management of the disease has an estimated cost of approximately 9.5 million euros per year only in the Pacific Northwest region, where half of the potato crops of the country are cultivated [3]. In Europe, CaLsol is mainly associated with Apiaceae crops [4,5,6]. The impact on carrot and other Apiaceae productions seems to be highly variable between the European regions [7], although it could give rise to severe yield losses [4,8] or losses of commercial value [9]. Since potato production is one of the most important horticultural crop systems in Europe (55.3 million tons in 2020) [10] and Spain (2.14 million tons in 2020) [11], the annual direct economic impact of an eventual infestation of solanaceous crops with CaLsol is estimated at 222 million euros [12], so the threat of ZC for European potato crops is serious. Naturally CaLsol-infected potato tubers have been reported with and without symptoms of ZC in Spain and Finland, respectively [13,14,15], but, so far, no studies have been carried out on the real incidence of this disease.

The characteristic symptom of ZC is a dark and light striped pattern in fresh plants and especially in chips, affecting their appearance and taste and making them commercially unacceptable. Foliar symptoms associated with ZC include upward rolling of the top leaves developing into a basal cupping of the leaflets, accompanied by yellowish or purplish discoloration, proliferation of axillary buds, shortened internodes, swollen nodes, aerial tubers, leaf chlorosis and scorching, and rapid plant death [1]. Symptoms in affected carrots include leaf curling; yellowish, bronze, and purplish discoloration of leaves; stunting of the shoots and roots; and proliferation of secondary roots [6].

The bacterium is transmitted by psyllid species belonging to the family Triozidae (Hemiptera: Psylloidea), which feed on plant phloem sap. The latent period of CaLsol in potato psyllid is approximately two weeks and the bacterium is transmitted in a persistent, circulative, and propagative manner [16,17].

Ten haplotypes of CaLsol have been described so far, causing damages in crops [7]. Haplotypes A and B are associated with diseases caused by the bacterium in potatoes and other Solanaceae species [18], and they are widely spread by *Bactericera cockerelli* (Šulc, 1909) throughout Central and North America and New Zealand. In Europe, haplotype C is associated with carrot and *Trioza apicalis* Foerster, 1848 in Finland [19]. Haplotypes D and E are described in carrots and other Apiaceae crops in the Mediterranean Basin [9,18], Spain [4,8,20], France [5], and North Africa [21]. In these production areas, CaLsol is transmitted by *Bactericera trigonica* Hodkinson, 1981 [22,23,24]. A sixth haplotype was found in the psyllid *Trioza urticae* (Linné, 1758) and stinging nettle (*Urtica dioica*, Urticaceae) in Finland, and named haplotype U [25]. A seventh haplotype has been identified, designated haplotype F, which is now the third member of this bacterium that has been found to infect potatoes in the United States [26]. Recently, two new haplotypes, G and H, have been detected. Haplotype G has been detected in samples of *Solanum umbelliferum* (Solanaceae) from Kern and Riverside counties (United States) from the University of California Riverside herbarium collection [27]. Haplotype H has been found and described in Apiaceae and Polygonaceae family plants from different regions of Finland [28].

Little is known about the role played by different psyllid species involved in CaLsol transmission, particularly in Europe, where different haplotypes, plant species, and vectors have been reported in the last few years. In particular, scarce information is available about the abundance and distribution of psyllid species in potato crops in Europe.

The presence of *B. trigonica* and *Bactericera nigricornis* (Foerster, 1848) in potato crops has been reported in Mainland Spain, and *B. trigonica* in the Canary Islands [20]. Moreover, Antolínez et al. [22] conducted surveys in potato and carrot crops in Mainland Spain in 2016 and 2017 during the cultivation cycle. These authors cited both species in carrot and potato crops, with *B. trigonica* being the most abundant in carrot and *B. nigricornis* the most abundant in potato. Antolínez et al. [29] considered that the risk of transmission of CaLsol from carrot to potato by *B. trigonica* seemed to be very limited. As *B. nigricornis* was found on both crops, this species could be responsible for the transmission of CaLsol from carrot to potato in Spain, since results of transmission experiments under controlled conditions showed that *B. nigricornis* was able to transmit CaLsol from carrot to potato plants [30]. Another species of *Bactericera* that could be involved in CaLsol transmission is *Bactericera tremblayi* (Wagner, 1961), the onion psyllid. This species was reported in a Spanish horticultural production area, causing important damages in leek crops [31], although it was stated as a carrier but not as a vector of CaLsol [29]. These three species are morphologically similar and belong to the *B. nigricornis* Foërster complex [32], but more extensive and systematic surveys are required to elucidate the actual role of each psyllid species in CaLsol transmission from carrot to potato crops.

In order to assess the real threat of CaLsol to potato production in Spain, regular and occasional surveys were carried out in large potato-producing areas of the country. The specific aims of this work were as follows: i, to determine the incidence of the disease in the main potato-producing areas, by detecting symptoms of the disease in plants and the bacterium by molecular methods; ii, to identify the psyllid species associated with potato crops in the main producing areas in Spain; and, iii, to study their abundance during the potato cropping season in order to determine the risk as a vector species of CaLsol.

## 2. Materials and Methods

### 2.1. Sampling Sites

**Figure 1:**
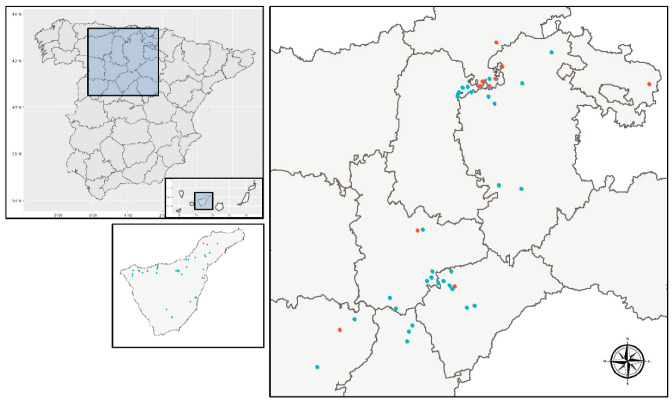
Locations of occasional (blue) and regular surveys (red) in Mainland Spain and the Canary Islands.

Occasional surveys. In total, 88 localities were visited: 44 between 2016 and 2018 in Mainland Spain (2016: 15; 2017: 15; 2018: 13) and 44 localities in the Canary Islands in 2016. Figure 1 shows sampling points for occasional surveys in Mainland Spain and in the Canary Islands. Monitoring for symptomatic plants was performed in occasional surveys. Twenty plants were collected per location in the mainland (≥1 ha was inspected per plot), and three plants in the Canary Islands (since the average size of the plots inspected was 0.1–0.2 ha). If plants did not have symptoms associated with zebra chip, asymptomatic plants were collected. Each sample consisted of three or four shoots from each plant, which were individually saved and kept at 4 °C until subjected to the laboratory procedure for DNA extraction. At the same time, potato canopies were sampled with a sweep net for psyllid identification and CaLsol detection in insects.

Regular surveys. Four different potato-growing areas in Spain were regularly surveyed throughout the growing season over three years (from 2016 to 2018), for monitoring the seasonal abundance of psyllid vectors (Figure 1). Potato-growing areas of Castile and Leon were monitored in three different locations, Aldearrubia (Salamanca), Gomezserracín (Segovia), and Zamadueñas (Valladolid), using different psyllid sampling methods: trapping by sticky yellow traps, sweeping, visual inspection of plants to look for psyllid eggs and nymphs (Gomezserracín and Zamadueñas in 2016), and horizontal green tile water traps (Zamadueñas 2017 and 2018). Several potato plots were monitored in the north of the Iberian Peninsula. Five potato fields were monitored by sticky yellow traps during the 2017 season in Cantabria, and three during the 2018 season, as well as another potato field by Moericke traps in Iturrieta, Euskadi (2016, 2017, 2018). Finally, in Tenerife (Canary Islands), during 2016, three potato plots were sampled in Güímar, Tegueste, and Valle de Guerra, over the cultivation cycle, using three different sampling methods: trapping by sticky yellow traps, visual inspection of plants, and horizontal yellow water traps. Insects captured by horizontal water traps and sweep nets were identified to species and gender level and then analyzed for CaLsol.

### 2.2. Sampling Methods for Insects

Several methods have been used for the detection and population monitoring of psyllids depending on convenience and with the aim of capturing all the species involved in most of the sampling periods.

Yellow sticky traps. These traps were sticky on both sides, 25 cm high and 10 cm wide, and were placed 0.5 m above ground on a wooden stake to trap adult psyllids (TA168, Sanidad Agrícola Econex, S.L., 30149 El Siscar, Murcia Spain). Sampling for adults began when the plants emerged and continued until the senescence of the canopy. Nine traps were placed in each plot, forming a square of 20 m size. Traps were collected from each field every 7–10 days, and psyllids on the sticky traps were observed under a binocular microscope and the number of specimens of *Bactericera* spp. was recorded.

Sweep net sampling. Potato canopies were sampled for adult psyllids using a telescopic folding sweep net. Each sample consisted of ten consecutive sweeps at ten different points, to obtain a total of ten samples per plot and date. Each sample was placed in a plastic zip-bag and stored in the laboratory at −20 °C for 48 h to kill the insects. Then, psyllids were selected and preserved in tubes with ethanol 70%, until sex and taxonomic identification and CaLsol analysis were performed.

Horizontal water traps. Moericke water traps (0.5 m × 0.5 m) were placed at canopy level, and one of them was located facing north and the other facing south at each sampling plot. Insects were recovered from the traps once a week. Insects obtained from Moericke water traps were preserved in 70% ethanol, which was then changed to 95% ethanol + 5% glycerol, and refrigerated.

Horizontal green tile water traps (also called Irwin traps) [33] were composed of methacrylate (16.5 cm × 16.5 cm × 4.5 cm) with a green-colored ceramic tile (15.5 cm × 15.5 cm) inside the container (Cambridge 815 from Cambridge tile C., PO Box 15071, Cincinnati, OH 46215, USA), filled with a 50% solution of ethylene glycol in water. The trap was placed at canopy level [22]. Collected insects were preserved in 70% ethanol until taxonomic identification.

Visual inspection of plants. To assess the developmental stages of psyllids, visual inspection of plants was performed to look for psyllid eggs and nymphs. Inspection was carried out from plant emergence to the senescence of the canopy. On each observation date, twenty plants were randomly selected in the plot, and the numbers of eggs and nymphs settled on each plant were counted. Nymphs were collected and reared in the laboratory until adult emergence for identification purposes (see below). The same procedure was also used in the occasional and regular surveys in the Canary Islands in 2016. Nymphs of stages 4 and 5 found on potato plants from regular surveys in Zamadueñas (2016) were taken with shoots, introduced in plastic tubes, and kept in the lab to allow their development at environmental conditions (17 tubes with at least one nymph/tube). Emerged psyllid adults reared in the laboratory from potato plants were identified as described below.

Morphological identification of psyllids. Intact psyllids collected with sweep net sampling and horizontal water traps were separated by gender and identified by morphological examination following the description of the species of the *Bactericera nigricornis* Foerster complex [31,32] using a Nikon SMZ1000 Zoom Stereo Microscope (Melville, NY 11747-3064, USA.).

### 2.3. CaLsol Detection in Psyllids and Plants

Preparation and DNA extraction of plant samples. Approximately 1 g of the plant sample was crushed into a Bioreba mesh plastic bag at 1:5–10 (wt/vol) in phosphate-buffered saline (PBS) extraction buffer (NaCl 8 g/l; NaH_2_PO_4_ 2H_2_O, 0.4 g/L; and Na_2_HPO_4_ 12H_2_O, 2.7 g/L; pH 7.2) with a Homex 6 homogenizer (Bioreba, 4153 Reinach, Switzerland). One ml from each sample was stored at –20 °C until use. DNA was purified from 200 µL of crude plant extract using a modification of the cetyltrimethyl ammonium bromide (CTAB) protocol without β-mercaptoethanol [34], as described in [7]. Purified DNA was preserved at −20 °C until use.

DNA extraction of psyllids. A non-destructive DNA extraction protocol using whole individual specimens was performed using the Chelex protocol [35]. Each specimen, with the head carefully separated from the body, was placed in a 1.5 mL Eppendorf tube, 10 µL of Proteinase K was added, and each tube was incubated at room temperature for 30 min. One hundred and fifty µL of 10% Chelex 100 resin solution in water was added to each tube; the tubes were sealed and incubated overnight at 55 °C. The mixture was centrifugated at 13,000× *g* for 3 min. The supernatant was extracted and the specimen was then removed and conserved in 70% ethanol for morphological identification. The material used during the preparation was cleaned and disinfected between specimens. The extracted DNA obtained was used for the CaLsol detections. The extracts were analyzed immediately or preserved at −20 °C until use.

Analysis for the detection of CaLsol was performed using the PlantPrint Detection Kit by Real-Time PCR (Taqman) using the conditions, primers, and probes described by [9]. This kit uses a master mix with all the components necessary for the amplification of the specific DNA (Taq polymerase, oligonucleotides, buffer, MgCl, primers, and TaqMan FAM/TAMRA probe). The PCR mix was prepared with 9 μL of master mix and 3 μL of total DNA of insects or plants, and amplification was performed using an initial cycle of 95 °C for 10 min and 45 cycles of 95 °C (15 s) and 64 °C (1 min). Controls supplied by the PlantPrint Kit were used as positive and negative controls.

## 3. Results

### 3.1. Occasional Surveys

#### 3.1.1. Detection of the Disease and the Bacterium in Plants

Eight hundred and eighty plants from 44 localities were analyzed in Mainland Spain in occasional surveys. During the first year of the study (2017), plants with unspecific symptoms were considered tentatively infected plants, but, in the following campaigns, only plants with more specific symptoms, such as dwarfism or aerial tubers, were selected as symptomatic. In all, 176 of the 880 plants analyzed showed symptoms such as deformed leaves (12%), yellowish leaves (6%), curling leaves (12%) (Figure S1), purple leaves (1%), chlorotic leaves (37%), plants with aerial tubers (4%), and dwarfism (28%). Only seven plants from two locations (Lomoviejo in 2016 and Remondo in 2017, both in Castile and Leon) tested positive for CaLsol (one in 2016 and six in 2017). Six of these plants showed aerial tubers (Remondo, 2017) and one plant (Lomoviejo, 2016) was asymptomatic (Table 1).

In the Canary Islands, 64 samples with symptoms, from 45 locations, were analyzed in occasional surveys. The proportion of symptoms among these samples was as follows: deformed leaves (36%), yellowish leaves (34%), curling leaves (18%), purple leaves (17%), chlorotic leaves (11%), plants with aerial tubers (6%), and dwarf plants (5%). None of the plants tested positive for CaLsol.

#### 3.1.2. Psyllid Species Associated with Potato Crop in Spain

In occasional surveys, 661 adult *Bactericera* psyllid specimens were captured in 36 out of 44 visited plots in Mainland Spain (Table S1 and Table 1). Specifically, 97% of psyllids captured by sweep nets were identified as *B. nigricornis* (639 out of 661). Only eighteen specimens of *B. trigonica* and four of *B. tremblayi* were captured, most of them in four potato fields located in Segovia province (Mozoncillo, Cogeces de Íscar, Chatún, and Remondo), where most of the carrot and leek crops are located in Spain, which also explains why the number of captured specimens was higher in Campo de Cuéllar (Segovia).

Specimens of *B. nigricornis* captured in occasional surveys showed low percentages of insects positive for CaLsol: only 12 out of 639 individuals yielded positive results (1.88%), and the bacterium was detected in 2 out of 18 captured insects identified as *B. trigonica* (Table 1).

### 3.2. Regular Surveys

#### 3.2.1. Study of the Abundance of the *Psyllid* Species during the Potato Cropping Season

In Mainland Spain, plots monitored with yellow sticky traps during the potato cropping season showed a high number of captured psyllids per trap (Figure S2A). On the plot located in Aldearrubia (Salamanca), population levels of psyllids were low throughout the season, reaching a peak in mid-June, and with an overall mean of 21.20 adults/trap. In Gomezserracín (Segovia), the potato plot showed higher levels of psyllids on traps, reaching the population peak in late August (684.67 ± 322.57 insects/trap). Finally, on the potato plot located in Zamadueñas (Valladolid), the peak psyllid population was higher (226.67 ± 38.71 insects/trap) and occurred later (in early July) than in Aldearrubia (Salamanca).

In Cantabria (north of the Iberian Peninsula), in 2017, we could identify three periods of time that showed population peaks on several field plots (Figure S2C). Firstly, some potato fields showed a population peak at the end of June (277 insects/trap in Cubillo de Ebro). Then, a second population peak was observed at the end of July (212 insects/trap in San Martín de Elines). Finally, a third population peak was observed at the end of August in San Martín de Elines (204 insects/trap). This pattern was reproduced in 2018, although with fewer catches (Figure S2D). It is necessary to highlight that, in these types of traps, it was not possible to distinguish the *Bactericera* species, but it was possible in other types of captures, such as sweep net sampling or water traps.

The most common species captured by sweep net sampling was *B. nigricornis* (Table 2). A total of 539 insects were captured at all sampling points throughout the study, of which 450 were identified as *B. nigricornis* and 89 as *B. trigonica*. Particularly in 2016, 71 insects identified as *B. trigonica* were trapped by this sampling method in Gomezserracín throughout the season, and 15 individuals of carrot psyllids were captured in Zamadueñas. These two plots were close to carrot crops, the main host plant for *B. trigonica*.

**Figure 2:**
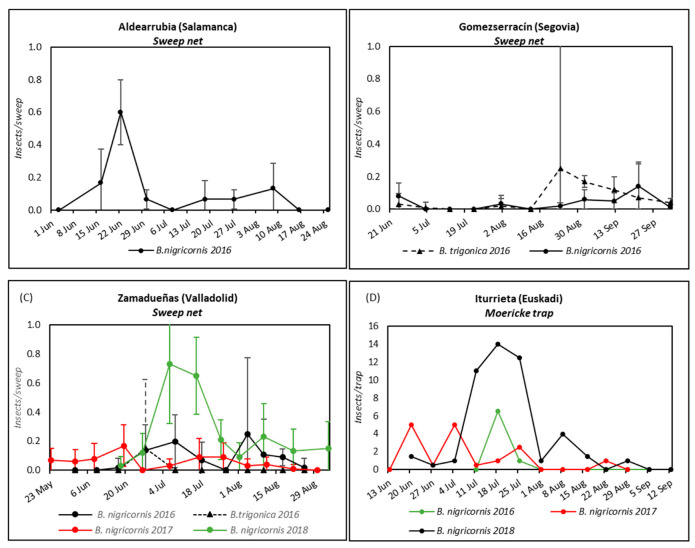
Mean number of psyllids collected per sweep net sampling in (**A**) Aldearrubia (Salamanca), (**B**) Gomezserracín (Segovia) and in (**C**) Zamadueñas (Valladolid); and per yellow Moericke water trap in (**D**) Iturrieta (Araba, Euskadi).

The weekly evolution of sweep net sampling captures in the field located in Aldearrubia (Salamanca), where only *B. nigricornis* adults were collected (see Figure 2A), showed that the first psyllids appeared in early June, reaching the maximum population peak at the end of this month (0.60 ± 0.20 psyllids/sweep); then, the psyllid population decreased and remained at low levels until the end of the season. In the field located in Gomezserracín (Segovia), both species had been collected, *B. nigricornis* and *B. trigonica*; the first psyllids appeared in late June; the *B. nigricornis* population level was low throughout the season and the *B. trigonica* population reached a small peak in late August (Figure 2B). On the plot located in Zamadueñas (Valladolid), psyllid population levels were also low during the whole season; the earliest appearance in potato fields occurred at 23 May 2017 (Figure 2C) and the maximum population peak in early July (0.73 ± 0.41 psyllids/sweep). In this location, *B. trigonica* was collected only in 2016 in late June and early July.

In Iturrieta (Araba, Euskadi), the number of specimens caught by Moericke traps was always very low (15 individuals in 2016, 31 in 2017, and 96 in 2018), with all of them being identified as *B. nigricornis* (Figure 2D). Captures were higher from mid-June to early September, with a maximum peak in mid-July.

A horizontal green water tile was installed in Zamadueñas (Valladolid) in 2017 and 2018 (Figure S3). No *B. trigonica* or *B. tremblayi* adults were captured during any season. No psyllid was captured in 2018, although a significant number of adults were captured by sweep net sampling. In 2017, a total of 25 psyllids were captured, all of them identified as *B. nigricornis* (Table 2). The largest number of captures (14 specimens) occurred at the beginning of the season (23 May 2017) and at the end of June (9 specimens).

**Figure 3:**
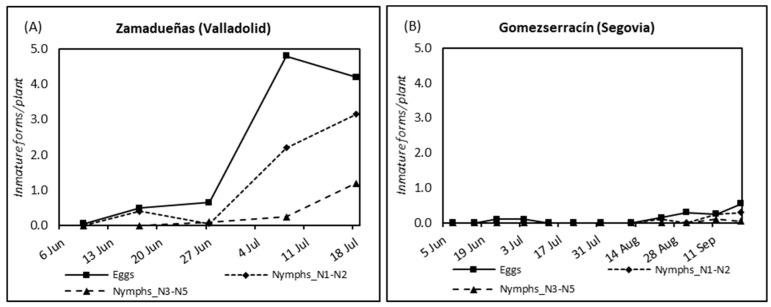
Mean of number of psyllid eggs per plant by visual inspection, number of nymphs per plant N1-N2, and nymphs per plant N3-N5 found in 2016 in (**A**) Zamadueñas (Valladolid) and (**B**) Gomezserracín (Segovia). X-axis represents collection dates and Y-axis values represent the mean number of eggs or nymphs per plant among twenty plants.

The numbers of eggs and nymphs counted by visual inspection in two potato fields are shown in Figure 3. In Gomezserracín (Segovia), the numbers of eggs and nymphs were low throughout the season, reaching a higher level at the end of the season in mid-September. In contrast, the numbers of eggs and immatures were higher in Zamadueñas (Valladolid), increasing drastically in early July, to reach the maximum number of eggs in 08 June 2016 (4.80 ± 0.22 eggs/plant), and the maximum number of nymphs N1-N2 ten days later (3.15 ± 2.64 nymphs/plant). Thirteen adults were obtained from nymphs reared in the lab, and all of them were identified as *B. nigricornis*.

In the Canary Islands, yellow sticky traps captured fewer psyllids of the *Bactericera* spp. (Figure S2B) than in Mainland Spain. Regarding horizontal water traps, 233 psyllids were captured, all of them were identified as *B. trigonica*, and *B. nigricornis* was not found (Table 2). Finally, neither psyllid eggs nor nymphs were observed in visual inspection in any of the visited potato fields. As *B. trigonica* cannot reproduce in potato [32], and no *B. nigricornis* specimen was found in horizontal water traps, we conclude that all *Bactericera* spp. captured by yellow sticky traps belonged to the *B. trigonica* species, and so we can state that *B. nigricornis* was not present in potato crops in the Canary Islands.

#### 3.2.2. Study of the Psyllids as Vector of CaLsol

The analysis for CaLsol in psyllids of the *B. nigricornis* species in regular surveys showed that the number of positive specimens was very low, both in males and females (Table 2), detecting the bacterium only in 14 males and 6 females out of 290 and 323 individuals, respectively, which constitutes 3.26% of positive insects overall (Table 2). On the contrary, the bacterium was detected in 267 of the 314 individuals identified as *B. trigonica*, reaching a percentage of 85.03% of CaLsol+ psyllids (Table 2), almost entirely captured in the Canary Islands. Although the rate of positive specimens of carrot psyllids was much higher than that of *B. nigricornis*, the presence of *B. trigonica* in potato crops was lower.

## 4. Discussion

CaLsol has been present in Europe for a long time; however, haplotype identification determined that American potato haplotypes (A and B), which cause zebra chip disease, are not reported in Europe [7], and no specimens of *B. cockerelli*, which is the CaLsol vector in America and New Zealand, have been found [1]. Otherwise, CaLsol is a major concern for potato growers in Europe since naturally CaLsol-infected potato tubers have already been reported in Spain and Finland [14,25]. Carrot CaLsol haplotypes have been identified in these positive potato samples (haplotypes C, D, and E) [15,25], but the distribution and prevalence in potato fields in Europe are still unknown.

The results of our survey showed very few CaLsol-positive plants in Mainland Spain, and many in the Canary Islands. Most of the symptoms described on potato plants were non-specific, finding, as a result, very few CaLsol+ symptomatic plants. Zebra-chip-like symptoms such as chlorotic, yellow, purple, curling, or deformed leaves; aerial stem tubers; and dwarf plants, alone or in combination, were mainly caused by potato leaf roll virus (PLRV), Rhizoctonia, or phytoplasmas when they were analyzed (data not shown). The aerial tubers were the most characteristic symptom of the disease, found in only one plot, since all the plants with this symptom were CaLsol+. Moreover, a single plant without any symptom but CaLsol+ was found, as reported in other production areas in Europe [25]. Symptom development depends on the cultivar tolerance and the period of CaLsol infection inside the plant, finding cultivars that develop symptoms from three to eight weeks after the moment of inoculation [36]. As the number of CaLsol+ plants detected was extremely low, we can consider that the incidence of the disease in potato crops in Spain is minimal at present.

It seems clear that any psyllid species could eventually transmit CaLsol from carrot to potato when plots of both crops are close in proximity. In spite of the high number of inspected fields, no *B. cockerelli* specimen was found in this study in any of the collected samples, neither in seasonal nor occasional surveys, regardless of the sampling method used. Therefore, the presence of the bacterium on potato fields in Spain (haplotypes D and E) [15] could potentially be due to any of the psyllids present in this crop. *B. nigricornis*, which has polyphagous habits, has been suggested as the vector responsible for CaLsol transmission from carrot to potato in Spain [22,30] and North Africa [23], since it has been found in both crops and shows a similar geographical distribution, although the capacity of this species to develop its entire life cycle in this crop had not been cited until now. Two other *Bactericera* species were tested as CaLsol vectors: *B. trigonica* and *B. tremblayi*. *B. trigonica* showed a very low transmission rate in potato [29], and *B. tremblayi* was able to acquire the bacterium when it was forced to feed on infected carrots, but no effective transmission to any crop was reported [29]. In order to evaluate the risk of the wide spreading of CaLsol in potato crops in Spain, we surveyed fields throughout the most important production areas.

*B. nigricornis* was found to be widely distributed in the northern half of the Iberian Peninsula; it was present in most of the visited potato plots in Castile and Leon, Euskadi, and Cantabria, but not in the Canary Islands. Moreover, *B. nigricornis* was the more frequently found psyllid species in potato crops in Mainland Spain, in accordance with the results obtained by Antolínez et al. [22], regardless of the method used for monitoring. The number of captured specimens of *B. trigonica* was higher than *B. nigricornis* only in Gomezserracín (Segovia), where the carrot crop is more important than potato, and the peak of *B. trigonica* captures matched with the most common period of carrot harvest; psyllids are widespread in this context, looking for new fields to feed on and colonize. *B. trigonica* was also abundantly caught by Moericke traps in the Canary Islands. Plots in Tenerife are small and potato plots are usually close to carrot ones, where *B. trigonica* is very abundant. Moreno et al. [30] reported that *B. nigricornis* was able to oviposit in potato under controlled conditions, but we detected immature forms of a psyllid species living on potato in Spain. In addition, when we reared immature forms of psyllids collected on potato fields in our laboratory, only adults of *B. nigricornis* were identified. Therefore, we can conclude that this psyllid species is the only one that could be a threat for potato crops, as plague or as a CaLsol vector, in Spain.

However, the number of captured psyllids was much lower than reported in other crops such as as carrot or celery in Europe [20,22] or potato in the USA [37], and it was similar to results reported by Antolínez et al. [22] for potato in Spain. The presence of *B. nigricornis* in Spanish potato fields was very scarce compared to other related psyllid species that cause serious damage to other crops, both by feeding on them and via the transmission of CaLsol. The maximum population peak reported in this study was 0.73 insects/sweep in Zamadueñas in regular surveys, while population peaks in *B. trigonica* in carrot were 35 insects/sweep in Íscar [22] and 3 insects/sweep for *B. cockerelli* in potato in the Northern USA [37]. Most of the insects captured by sweep nets in Zamadueñas were identified as *B. nigricornis*, which suggests that the peak of 227 individuals/yellow sticky trap every 10 days would match mostly with this species. These values do not differ significantly from those obtained by Nissinen et al. [38] for *T. apicalis* in carrot (100 specimens/trap and week), but they are far from the levels of 500 individuals/trap and week reported by Antolínez et al. [22], or the 300 individuals per week obtained for *B. cockerelli* in potato in the Northern USA [37].

In addition, in regular surveys, *B. nigricornis* remained during the whole crop cycle, variable depending on the year and the location but generally when the plant presented higher foliage during the months of June and July, in the conditions of the Northern Mainland. From the month of August, when the senescence of the crop starts, the population of psyllids decreases.

Less than 10% of the *B. cockerelli* field population in the United States were found to carry CaLsol [39]; however, the proportion of the *T. apicalis* population harboring CaLsol in Finland was 61% to 67% [6,40]. In our study, CaLsol+ insect percentages varied between 1.88 and 3.90% in *B. nigricornis* and 33.33% and 85.03% in *B. trigonica*. Although carrot psylla showed sufficient levels of bacteria-carrying insects, it was reported that the risk of CaLsol transmission mediated by *B. trigonica* in potato was very low [29]. Regarding *B. nigricornis*, despite the low number of CaLsol+ captured specimens, it has been reported that it is able to transmit CaLsol to potato plants, although in a less efficient manner than in carrot [30].

Several methods have been used for the detection and population monitoring of psyllids, such as suction traps, vacuum sampling of plants, sweep net sampling, visual inspection of plant material, horizontal water traps, and colored sticky traps. Suction traps and vacuum samplers were found to be ineffective for detecting and sampling *B. cockerelli*, respectively [39,41]. However, sweep nets and sticky card traps are used extensively [37,39,41]. These methods, sweeping and trapping by yellow sticky cards, were also used for *B. trigonica* and *B. nigricornis* [22,23]. Moreover, visual inspection provided detailed information regarding the population density of these pests and was used for *B. cockerelli* [37,39,41], *B. trigonica* [22,23], and *B. nigricornis* [23] population monitoring. Water horizontal traps were used for determining which species were landing over the crop, looking for a plant to feed on [33]. This tool was used in carrot and potato by Antolínez et al. [22], being effective to catch the early immigrants. In our study, monitoring by sweeping net was very effective; samples could be identified at species level and subsequently CaLsol could be analyzed in insects. However, a long training time is necessary to carry out the identification. Yellow sticky traps have been widely used since they are very useful for detecting adult population peaks and are easy to manage, although individuals cannot be identified to species level, but only genus level. Further, these traps exert attraction for insects, collecting individuals that are not properly found in the sampling plot. On the other hand, visual inspection provides detailed information about the population density of immature forms of these insects in plants, allowing us to identify the moment of the initial appearance of the pest in the crop. Furthermore, in this work, thanks to visual inspection, it was possible to observe, for the first time, the development of *B. nigricornis* in potato crops in Spain, where this insect has managed to complete the cycle. The results show that the psyllid *B. nigricornis* is not a threat species in potato crops, regardless of the capture method used.

## 5. Conclusions

Although CaLsol haplotypes in Europe are different from American ones and some epidemiological differences could exist, the abundance of *B. nigricornis* does not seem sufficient to pose a threat for potato production, due to the scarce number of specimens and because the frequency of *B. nigricornis* specimens that are CaLsol+ is very low. The maximum population of this species matched with the vegetative growth of potato plants, the optimum moment of CaLsol infection, and suitable temperatures for ZC symptom development. These conditions allow the presence of ZC symptoms in harvesting tubers.

## Figures and Tables

**Table 1:** Symptomatic and positive plants for *Candidatus* Liberibacter solanacearum (CaLsol); mean and number of males and females and percentage of CaLsol+ specimens of *Bactericera nigricornis* captured by sweep net, in occasional surveys in potato fields, in different production areas, in 2016, 2017, and 2018.

Locality	Province	Region	Date	Symptomatic Plants	CaLsol+	*Bactericera Nigricornis*					
						Insects/Sweep		♂		♀	
						Mean	St Dv	N	CaLsol+	N	CaLsol+
Cogeces de Íscar	Valladolid	Castile and Leon	28 June 2016	13	0	0.19	0.19	9	0	10	0
Íscar	Valladolid	Castile and Leon	28 June 2016	10	0	0.31	0.44	0	-	14	0
Pedrajas de San Esteban	Valladolid	Castile and Leon	28 June 2016	17	0	0.04	0.13	0	-	4	0
Escalona del Prado	Segovia	Castile and Leon	30 June 2016	19	0	0.00	0.00	0	-	0	-
Mozoncillo	Segovia	Castile and Leon	30 June 2016	7	0	0.34	0.22	18	6	16	0
Torregutiérrez	Segovia	Castile and Leon	30 June 2016	10	0	0.47	0.18	14	0	33	0
Cabizuela	Ávila	Castile and Leon	7 July 2016	7	0	0.00	0.00	0	-	0	-
Nava de Arévalo	Ávila	Castile and Leon	7 July 2016	13	0	0.02	0.04	2	0	0	-
Vinaderos	Ávila	Castile and Leon	7 July 2016	11	0	0.04	0.07	0	-	4	2
Quintanilla del Agua	Burgos	Castile and Leon	21 July 2016	19	0	0.01	0.03	0	-	1	0
Tordomar	Burgos	Castile and Leon	21 July 2016	0	0	0.04	0.07	0	-	4	0
Cantalpino	Salamanca	Castile and Leon	28 July 2016	19	0	0.02	0.06	0	-	2	0
Pedrosillo de los Aires	Salamanca	Castile and Leon	28 July 2016	17	0	0.07	0.13	3	0	4	0
Cabezón de Pisuerga	Valladolid	Castile and Leon	9 August 2016	3	0	0.01	0.03	0	0	1	0
Lomoviejo	Valladolid	Castile and Leon	9 August 2016	0	1	0.05	0.16	2	-	3	-
Velascálvaro	Valladolid	Castile and Leon	9 August 2016	4	0	0.10	0.32	3	0	7	0
Chatún	Segovia	Castile and Leon	1 August 2017	0	0	0.04	0.05	3	0	2	0
Cogeces de Íscar	Segovia	Castile and Leon	1 August 2017	0	0	0.22	0.20	10	0	12	0
Remondo	Segovia	Castile and Leon	1 August 2017	6	6	0.05	0.05	1	0	4	0
Castresana de Losa	Burgos	Castile and Leon	3 August 2017	0	0	0.00	0.00	0	-	0	-
Dobro	Burgos	Castile and Leon	3 August 2017	0	0	0.00	0.00	0	-	0	-
Fuenteurbel	Burgos	Castile and Leon	3 August 2017	0	0	0.07	0.08	0	-	7	1
Cubillo de Ebro	Santander	Cantabria	17 August 2017	0	0	0.00	0.00	0	-	0	-
Montecillo	Santander	Cantabria	17 August 2017	0	0	0.22	0.18	3	0	19	0
Renedo de Bricia	Santander	Cantabria	17 August 2017	0	0	0.02	0.06	1	0	1	0
San Martín de Elines	Santander	Cantabria	17 August 2017	0	0	0.00	0.00	0	-	0	-
Villamoñico	Santander	Cantabria	17 August 2017	0	0	0.00	0.00	0	-	0	-
Fuencaliente de Valdelucio	Burgos	Castile and Leon	17 August 2017	0	0	0.19	0.19	4	0	15	1
Becerril de Carpio	Palencia	Castile and Leon	17 August 2017	0	0	0.04	0.07	3	1	1	0
Santa Mª de Mave	Palencia	Castile and Leon	17 August 2017	0	0	0.27	0.25	11	0	16	0
Villallano	Palencia	Castile and Leon	17 August 2017	0	0	0.47	0.13	18	0	29	0
Chañe	Segovia	Castile and Leon	12 July 2018	0	0	0.16	0.16	4	0	12	0
Remondo	Segovia	Castile and Leon	12 July 2018	0	0	0.27	0.25	6	0	21	0
Cogeces de Íscar	Valladolid	Castile and Leon	12 July 2018	0	0	0.03	0.05	0	-	3	0
Cubillo	Santander	Cantabria	18 July 2018	0	0	0.02	0.06	0	-	2	-
Campo de Cuéllar	Segovia	Castile and Leon	24 July 2018	0	0	2.26	0.73	66	1	160	0
Ruerrero	Santander	Cantabria	30 July 2018	0	0	0.10	0.11	2	-	8	-
Espinosa	Santander	Cantabria	7 August 2018	0	0	0.08	0.08	2	-	6	-
Cubillo	Santander	Cantabria	21 August 2018	0	0	0.04	0.10	0	-	4	-
Basconcillos del Tozo	Burgos	Castile and Leon	21 August 2018	0	0	0.07	0.11	2	0	5	0
Santa Mª Mave	Palencia	Castile and Leon	21 August 2018	0	0	0.18	0.28	5	0	13	0
Villarén de Valdivia	Palencia	Castile and Leon	21 August 2018	0	0	0.03	0.07	2	0	1	0
Ruerrero	Santander	Cantabria	6 September 2018	0	0	0.00	0.00	0	-	0	-
Espinosa	Santander	Cantabria	13 September 2018	-	-	0.01	0.03	0	-	1	-
Total					7			194	8	445	4

**Table 2:** Number of psyllids by sampling tool, identified by species and gender, and CaLsol detecTable 2016, 2017, and 2018.

Locality	Province	Region (1)	Year	Sampling Tool (2)	*Bactericera Nigricornis*					*Bactericera Trigonica*				
					Total	♂	CaLsol+	♀	CaLsol+	Total	♂	CaLsol+	♀	CaLsol+
Güimar	Tenerife	CI	2016	MT	0	0	-	0	-	152	139	136	5	5
Isamar	Tenerife	CI	2016	MT	0	0	-	0	-	17	13	8	4	0
Tegueste	Tenerife	CI	2016	MT	0	0	-	0	-	64	48	45	16	16
Aldearrubia	Salamanca	CyL	2016	SN	33	11	1	22	0	0	0	-	0	-
Gomezserracín	Segovia	CyL	2016	SN	39	11	1	28	2	71	51	34	20	15
Zamadueñas	Valladolid	CyL	2016	SN	88	26	0	62	0	15	8	4	7	3
Iturrieta	Araba	Euskadi	2016	MT	15	10	2	5	0	0	0	-	0	-
Zamadueñas	Valladolid	CyL	2017	IT	23	14	6	9	2	0	0	-	0	-
Zamadueñas	Valladolid	CyL	2017	SN	64	17	0	45	0	2	0	-	2	1
Iturrieta	Araba	Euskadi	2017	MT	31	26	2	5	0	0	0	-	0	-
Zamadueñas	Valladolid	CyL	2018	IT	0	0	-	0	-	0	0	-	0	-
Zamadueñas	Valladolid	CyL	2018	SN	226	91	0	135	2	1	0	-	1	0
Iturrieta	Araba	Euskadi	2018	MT	96	84	2	12	0	0	0	-	0	-
Total					615	290	14	323	6	322	259	227	55	40

(1) CI = Canary Islands, CyL = Castile and Leon; (2) MT = Moericke Trap, SN = Sweep Net, IT = Irwin Trap.

## Data Availability

Data is contained within the article or Supplementary Material.

## Supplementary Materials

The following supporting information can be downloaded at: https://www.mdpi.com/article/10.3390/insects13100964/s1, Figure S1: Symptoms found in potato plants in occasional surveys in Mainland Spain; Figure S2: Mean of number of psyllids per yellow sticky trap in (A) Castile and Leon in 2016; (B) Tenerife (Canary Islands) in 2016; (C) Cantabria in 2017; and (D) Cantabria in 2018; Figure S3: Mean of number of psyllids captured by Irwin traps in Zamadueñas (Valladolid, Castilla, and León) in 2017; Table S1: Symptomatic and positive plants for CaLsol; mean and number of males and females and percentage of CaLsol+ specimens of *Bactericera* species captured by sweep net on occasional surveys in potato fields in different production areas in 2016, 2017, and 2018.
