# Supplementary material for: Survey of Candidatus Liberibacter Solanacearum and Its Associated Vectors in Potato Crop in Spain
Source: Insects. 2022 Oct 21;13(10):964. doi: 10.3390/insects13100964 (PMC9604363; doi:10.3390/insects13100964)
Supplement: Supplementary file 1 [file insects-13-00964-s001.zip › Figure S2.pdf]

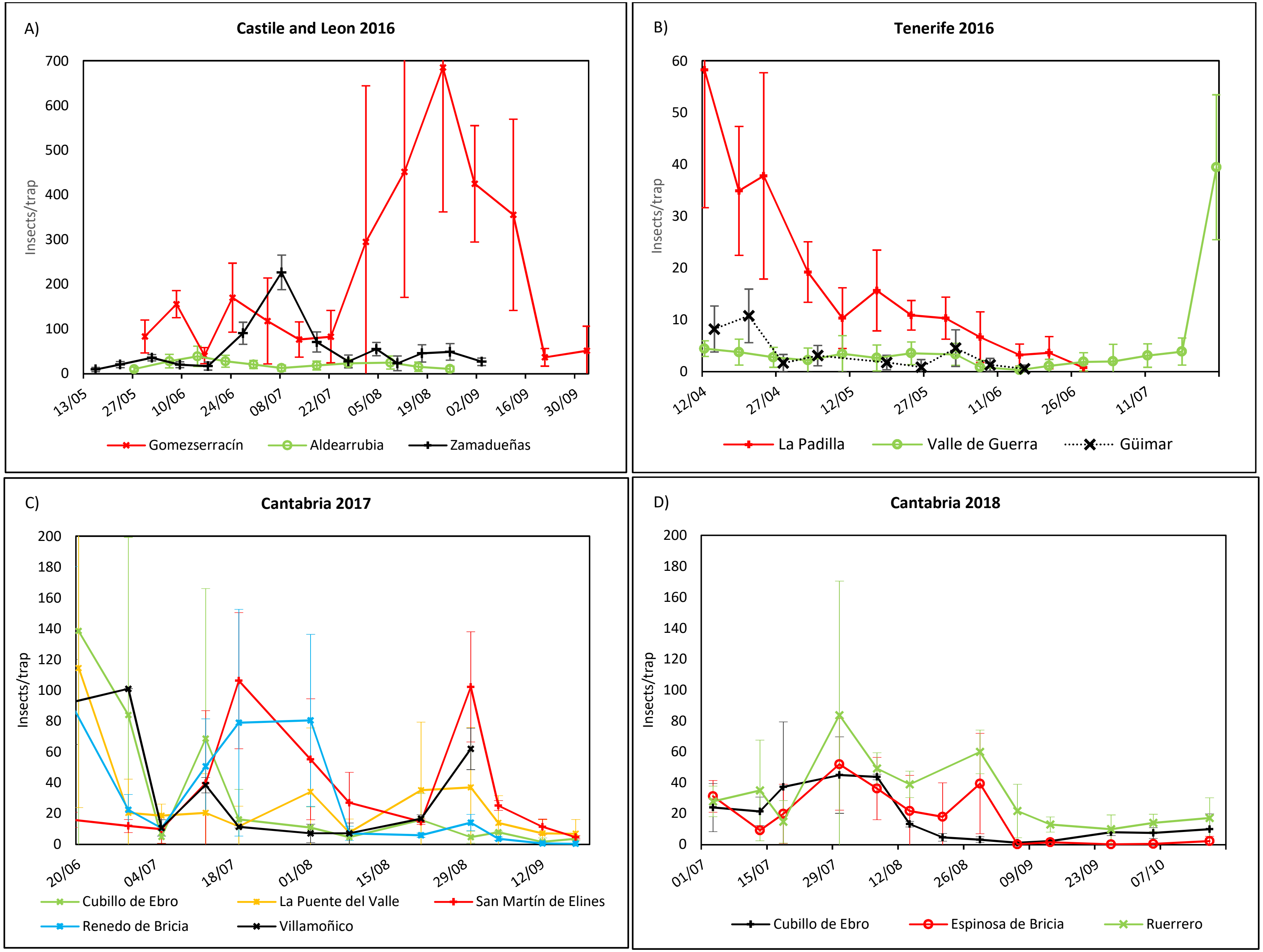

Figure S2. Mean of number of psyllids per yellow sticky trap in A) Castile and Leon in 2016; B) Tenerife (Canary Islands) in 2016; C) Cantabria in 2017; and D) Cantabria in 2018.
