# Supplementary material for: Survey of Candidatus Liberibacter Solanacearum and Its Associated Vectors in Potato Crop in Spain
Source: Insects. 2022 Oct 21;13(10):964. doi: 10.3390/insects13100964 (PMC9604363; doi:10.3390/insects13100964)
Supplement: Supplementary file 1 [file insects-13-00964-s001.zip › Figure S3.pdf]

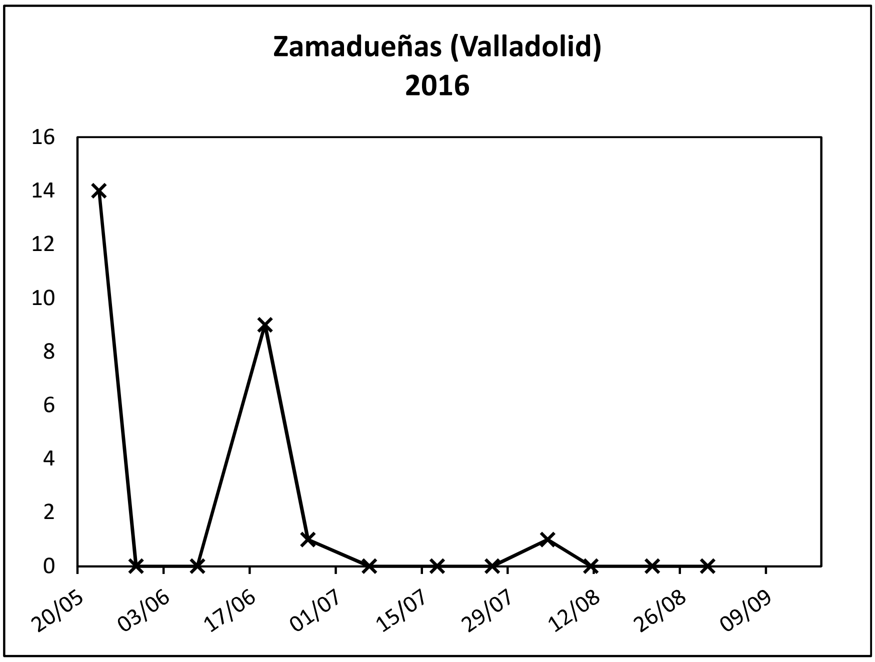

Figure S3. Mean of number of psyllids captures by Irwin traps in Zamadueñas (Valladoli, Castilla y León) in 2017.
