# Supplementary material for: Survey of Candidatus Liberibacter Solanacearum and Its Associated Vectors in Potato Crop in Spain
Source: Insects. 2022 Oct 21;13(10):964. doi: 10.3390/insects13100964 (PMC9604363; doi:10.3390/insects13100964)
Supplement: Supplementary file 1 [file insects-13-00964-s001.zip › Table S1.pdf]

Table S1. Symptomatic and positive plants for CaLsol; mean and number of males and females and percentage of CaLsol+ specimens of *Bactericera* species captured by sweep net on occasional surveys in potato fields in different production areas in 2016, 2017 and 2018.

						B. nigricornis						***B. trigonica***						***B. tremblayi***					
				**Symptomatic**		**insects/sweep**		**♂**		**♀**		**insects/sweep**		**♂**		**♀**		**insects/sweep**		**♂**		**♀**	
**Locality**	**Province**	**Region**	**Date**	**plants**	**CaLsol+**	**mean**	**dv st**	**N**	**CaLsol+**	**N**	**CaLsol+**	**mean**	**dv st**	**N**	**CaLsol+**	**N**	**CaLsol+**	**mean**	**dv st**	**N**	**CaLsol+**	**N**	**CaLsol+**
Cogeces de Íscar	Valladolid	Castile and Leon	28/06/2016	13	0	0.19	0.19	9	0	10	0	0.00	0.00	0	-	0	-	0.00	0.00	0	-	0	-
Íscar	Valladolid	Castile and Leon	28/06/2016	10	0	0.31	0.44	0	-	14	0	0.00	0.00	0	-	0	-	0.00	0.00	0	-	0	-
Pedrajas de San Esteban	Valladolid	Castile and Leon	28/06/2016	17	0	0.04	0.13	0	-	4	0	0.00	0.00	0	-	0	-	0.00	0.00	0	-	0	-
Escalona del Prado	Segovia	Castile and Leon	30/06/2016	19	0	0.00	0.00	0	-	0	-	0.00	0.00	0	-	0	-	0.00	0.00	0	-	0	-
Mozoncillo	Segovia	Castile and Leon	30/06/2016	7	0	0.34	0.22	18	6	16	0	0.01	0.03	1	0	0	-	0.00	0.00	0	-	0	-
Torregutiérrez	Segovia	Castile and Leon	30/06/2016	10	0	0.47	0.18	14	0	33	0	0.00	0.00	0	-	0	-	0.00	0.00	0	-	0	-
Cabizuela	Ávila	Castile and Leon	07/07/2016	7	0	0.00	0.00	0	-	0	-	0.00	0.00	0	-	0	-	0.00	0.00	0	-	0	-
Nava de Arévalo	Ávila	Castile and Leon	07/07/2016	13	0	0.02	0.04	2	0	0	-	0.00	0.00	0	-	0	-	0.00	0.00	0	-	0	-
Vinaderos	Ávila	Castile and Leon	07/07/2016	11	0	0.04	0.07	0	-	4	2	0.00	0.00	0	-	0	-	0.00	0.00	0	-	0	-
Quintanilla del Agua	Burgos	Castile and Leon	21/07/2016	19	0	0.01	0.03	0	-	1	0	0.00	0.00	0	-	0	-	0.00	0.00	0	-	0	-
Tordomar	Burgos	Castile and Leon	21/07/2016	0	0	0.04	0.07	0	-	4	0	0.00	0.00	0	-	0	-	0.00	0.00	0	-	0	-
Cantalpino	Salamanca	Castile and Leon	28/07/2016	19	0	0.02	0.06	0	-	2	0	0.00	0.00	0	-	0	-	0.00	0.00	0	-	0	-
Pedrosillo de los Aires	Salamanca	Castile and Leon	28/07/2016	17	0	0.07	0.13	3	0	4	0	0.00	0.00	0	-	0	-	0.00	0.00	0	-	0	-
Cabezón de Pisuerga	Valladolid	Castile and Leon	09/08/2016	3	0	0.01	0.03	0	0	1	0	0.00	0.00	0	-	0	-	0.00	0.00	0	-	0	-
Lomoviejo	Valladolid	Castile and Leon	09/08/2016	0	1	0.05	0.16	2	-	3	-	0.00	0.00	0	-	0	-	0.00	0.00	0	-	0	-
Velascálvaro	Valladolid	Castile and Leon	09/08/2016	4	0	0.10	0.32	3	0	7	0	0.00	0.00	0	-	0	-	0.00	0.00	0	-	0	-
Chatún	Segovia	Castile and Leon	01/08/2017	0	0	0.04	0.05	3	0	2	0	0.02	0.04	1	0	1	1	0.04	0.07	0	-	2	0
Cogeces de Íscar	Segovia	Castile and Leon	01/08/2017	0	0	0.22	0.20	10	0	12	0	0.03	0.05	0	-	3	1	0.00	0.00	0	-	0	-
Remondo	Segovia	Castile and Leon	01/08/2017	6	6	0.05	0.05	1	0	4	0	0.00	0.00	0	-	0	-	0.02	0.04	0	-	1	0
Castresana de Losa	Burgos	Castile and Leon	03/08/2017	0	0	0.00	0.00	0	-	0	-	0.00	0.00	0	-	0	-	0.00	0.00	0	-	0	-
Dobro	Burgos	Castile and Leon	03/08/2017	0	0	0.00	0.00	0	-	0	-	0.00	0.00	0	-	0	-	0.00	0.00	0	-	0	-
Fuenteurbel	Burgos	Castile and Leon	03/08/2017	0	0	0.07	0.08	0	-	7	1	0.00	0.00	0	-	0	-	0.00	0.00	0	-	0	-
Cubillo de Ebro	Santander	Cantabria	17/08/2017	0	0	0.00	0.00	0	-	0	-	0.00	0.00	0	-	0	-	0.00	0.00	0	-	0	-
Montecillo	Santander	Cantabria	17/08/2017	0	0	0.22	0.18	3	0	19	0	0.00	0.00	0	-	0	-	0.00	0.00	0	-	0	-
Renedo de Bricia	Santander	Cantabria	17/08/2017	0	0	0.02	0.06	1	0	1	0	0.00	0.00	0	-	0	-	0.00	0.00	0	-	0	-
San Martín de Elines	Santander	Cantabria	17/08/2017	0	0	0.00	0.00	0	-	0	-	0.00	0.00	0	-	0	-	0.00	0.00	0	-	0	-
Villamoñico	Santander	Cantabria	17/08/2017	0	0	0.00	0.00	0	-	0	-	0.00	0.00	0	-	0	-	0.00	0.00	0	-	0	-
Fuencaliente de Valdelucio	Burgos	Castile and Leon	17/08/2017	0	0	0.19	0.19	4	0	15	1	0.00	0.00	0	-	0	-	0.00	0.00	0	-	0	-
Becerril de Carpio	Palencia	Castile and Leon	17/08/2017	0	0	0.04	0.07	3	1	1	0	0.00	0.00	0	-	0	-	0.00	0.00	0	-	0	-
Santa Mª de Mave	Palencia	Castile and Leon	17/08/2017	0	0	0.27	0.25	11	0	16	0	0.00	0.00	0	-	0	-	0.00	0.00	0	-	0	-
Villallano	Palencia	Castile and Leon	17/08/2017	0	0	0.47	0.13	18	0	29	0	0.00	0.00	0	-	0	-	0.00	0.00	0	-	0	-
Chañe	Segovia	Castile and Leon	12/07/2018	0	0	0.16	0.16	4	0	12	0	0.00	0.00	0	-	0	-	0.00	0.00	0	-	0	-
Remondo	Segovia	Castile and Leon	12/07/2018	0	0	0.27	0.25	6	0	21	0	0.00	0.00	0	-	0	-	0.00	0.00	0	-	0	-
Cogeces de Íscar	Valladolid	Castile and Leon	12/07/2018	0	0	0.03	0.05	0	-	3	0	0.00	0.00	0	-	0	-	0.02	0.04	0	-	1	0
Cubillo	Santander	Cantabria	18/07/2018	0	0	0.02	0.06	0	-	2	-	0.00	0.00	1	-	1	-	0.00	0.00	0	-	0	-
Campo de Cuéllar	Segovia	Castile and Leon	24/07/2018	0	0	2.26	0.73	66	1	160	0	0.00	0.00	0	-	0	-	0.00	0.00	0	-	0	-
Ruerrero	Santander	Cantabria	30/07/2018	0	0	0.10	0.11	2	-	8	-	0.02	0.04	1	-	1	-	0.00	0.00	0	-	0	-
Espinosa	Santander	Cantabria	07/08/2018	0	0	0.08	0.08	2	-	6	-	0.10	0.28	0	-	8	-	0.00	0.00	0	-	0	-
Cubillo	Santander	Cantabria	21/08/2018	0	0	0.04	0.10	0	-	4	-	0.00	0.00	0	-	0	-	0.00	0.00	0	-	0	-
Basconcillos del Tozo	Burgos	Castile and Leon	21/08/2018	0	0	0.07	0.11	2	0	5	0	0.00	0.00	0	-	0	-	0.00	0.00	0	-	0	-
Santa Mª Mave	Palencia	Castile and Leon	21/08/2018	0	0	0.18	0.28	5	0	13	0	0.00	0.00	0	-	0	-	0.00	0.00	0	-	0	-
Villarén de Valdivia	Palencia	Castile and Leon	21/08/2018	0	0	0.03	0.07	2	0	1	0	0.00	0.00	0	-	0	-	0.00	0.00	0	-	0	-
Ruerrero	Santander	Cantabria	06/09/2018	0	0	0.00	0.00	0	-	0	-	0.00	0.00	0	-	0	-	0.00	0.00	0	-	0	-
Espinosa	Santander	Cantabria	13/09/2018	-	-	0.01	0.03	0	-	1	-	0.00	0.00	0	-	0	-	0.00	0.00	0	-	0	-
Total					7			194	8	445	4			4	0	14	2			0	0	4	0
