# Supplementary figures and images for: Survey of Candidatus Liberibacter Solanacearum and Its Associated Vectors in Potato Crop in Spain
Source: Insects. 2022 Oct 21;13(10):964. doi: 10.3390/insects13100964 (PMC9604363; doi:10.3390/insects13100964)

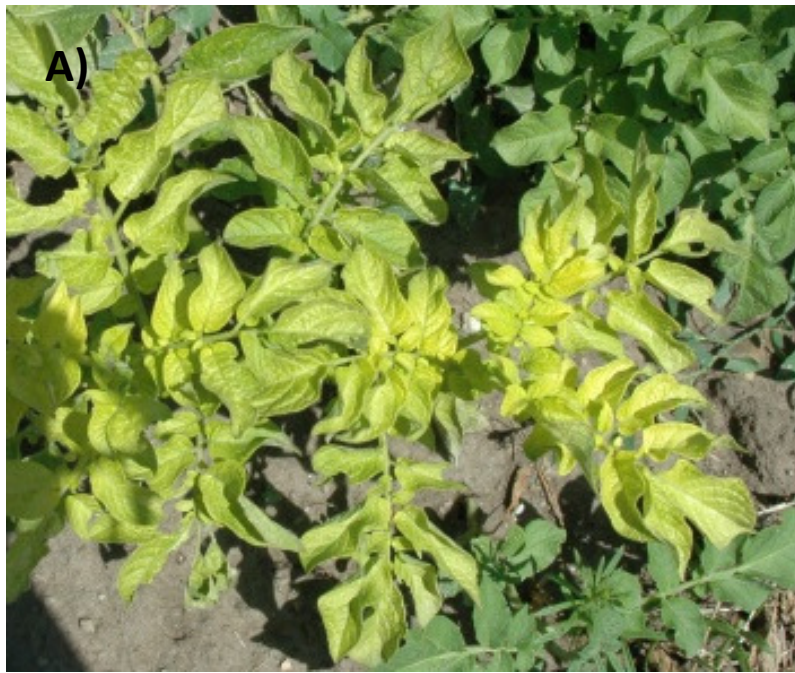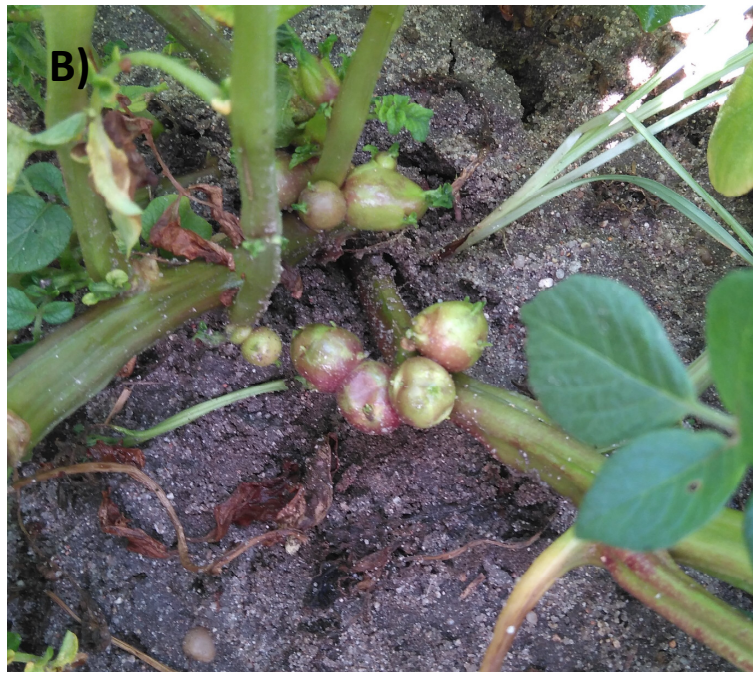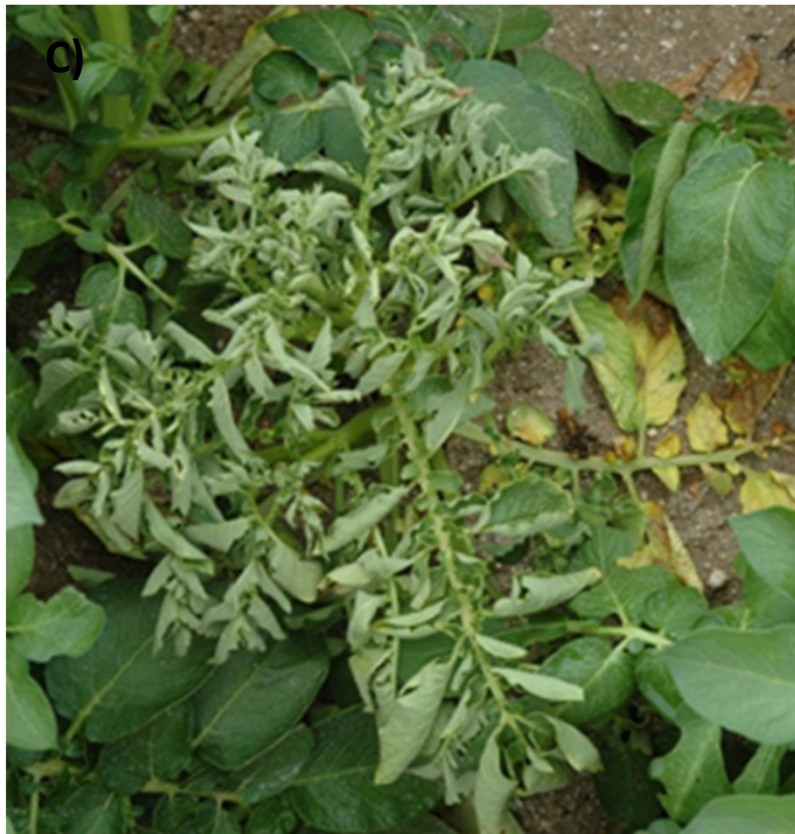

**D)**

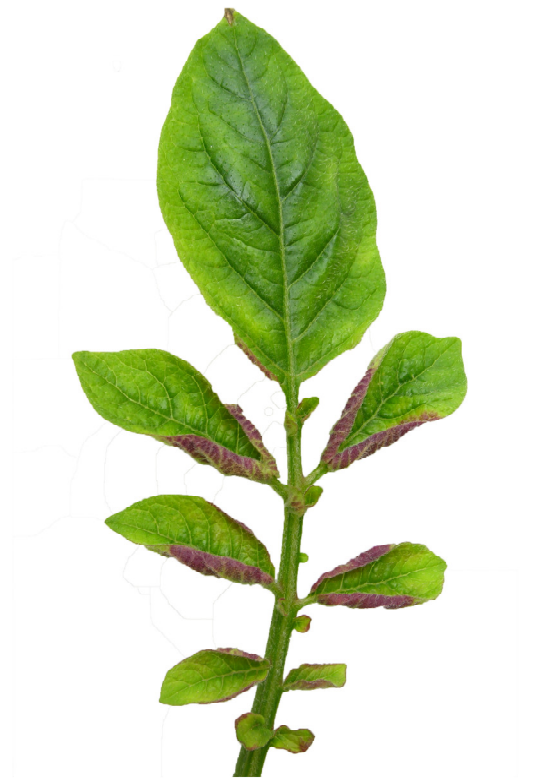

Figure S1. Symptoms found in potato plants in occasional surveys in Mainland Spain.

Supplement: Supplementary file 1 [file insects-13-00964-s001.zip › Figure S1.pdf]
